# Awareness of Forensic Odontology among Dental Students and Faculty in Cyprus: A Survey-Based Study

**DOI:** 10.3390/dj12010006

**Published:** 2023-12-26

**Authors:** Kostis Giannakopoulos, Persefoni Lambrou-Christodoulou, Eleftherios G. Kaklamanos

**Affiliations:** 1 School of Dentistry, European University Cyprus, Nicosia 2404, Cyprus; p.lambrou@external.euc.ac.cy (P.L.-C.); e.kaklamanos@external.euc.ac.cy (E.G.K.); 2 School of Dentistry, Aristotle University of Thessaloniki, 54124 Thessaloniki, Greece; 3 Hamdan Bin Mohammed College of Dental Medicine, Mohammed Bin Rashid University of Medicine and Health Sciences, Dubai 505055, United Arab Emirates

**Keywords:** forensic odontology, forensic dentistry, awareness on forensic odontology, dental students, dental faculty, Cyprus, European Union, European University Cyprus

## Abstract

This study aimed to evaluate the awareness, comprehension, and practices concerning forensic odontology among dental students and faculty at a Dental School in Cyprus. An online, cross-sectional, descriptive survey, employing an adapted, self-administered questionnaire, was disseminated to all dental students and faculty at the School of Dentistry, European University Cyprus, in November 2022. The survey assessed participants’ demographic information and explored their awareness with questions alluding to knowledge, attitudes and practices in forensic dentistry. Of those surveyed, 47 faculty members and 304 students responded, yielding response rates of 66.2% and 80%, respectively. Statistical analysis, including Kendall’s *tau* test and χ^2^ test were employed to examine correlations and associations, with Cramer’s V used to measure the strength of significant associations. The predetermined significance level was α = 0.05. Awareness levels were assessed through participants’ responses to specific questions in the survey. It was revealed that 87% of faculty and 65% of students were familiar with forensic odontology. A noteworthy 94% of faculty and 85% of students recognized teeth as DNA repositories. A high percentage, 98% of faculty and 89% of students, acknowledged the role of forensic odontology in the identification of criminals and deceased individuals. Awareness of age estimation through dental eruption patterns was evident in 85% of faculty and 81.6% of students. A substantial proportion (80% of faculty) maintained dental records, while 78% of students recognized the importance of dental record-keeping in ensuring quality care. Interestingly, 57% of students and 64% of faculty were aware of the possibility of dentists testifying as expert witnesses. The majority, 95.7% of faculty and 85% of students, concurred that physical harm, scars, and behavioral alterations predominantly indicate child abuse. The findings, revealing robust awareness among respondents, underscore the importance of enhancing faculty engagement in relevant seminars to further strengthen their knowledge. Additionally, emphasizing improved record-keeping practices for potential forensic applications emerges as a crucial aspect. These insights have implications for refining dental education in Cyprus and enhancing forensic practices by promoting ongoing professional development and emphasizing meticulous record-keeping within the dental community.

## 1. Introduction

Forensic Odontology constitutes a rapidly developing branch of forensic science, with immense importance in the examination of forensic dental evidence in legal circumstances and the identification of victims of mass disasters or abuse [1,2]. Forensic odontology utilizes information from many dental disciplines (oral surgery, radiology, restorative dentistry, orthodontics, etc.) [3] and is primarily concerned with its use in legal contexts [4,5,6]. In modern times, forensic odontologists have become invaluable members of forensic investigation teams [6,7,8].

Due to the enduring nature of hard oral tissues, forensic dentists play a crucial role in human identification, leveraging the uniqueness of dental morphology. This is particularly valuable even in cases of severe body damage, as each individual’s oral cavity is distinct, ensuring that no two sets of dentition are alike [9,10]. Dental features, encompassing aspects like tooth morphology, variations in shape and size, restorations, pathologies, missing teeth, wear patterns, color, and the position of teeth (including crowding and rotations), among other characteristics, contribute to an individual’s unique oral identity [11]. The comparison of ante- and post-mortem dental records remains one of the most effective and commonly employed methods in forensic odontology [12]. In the absence of latter, teeth can help in the determination of age, biological sex, population affinities, habits and occupation, which can serve as additional clues regarding the identity of individuals [13].

Dental schools offer students the chance to understand the significance of maintaining thorough and precise dental records, a crucial aspect of ensuring quality patient care and adhering to sound clinical practice. Moreover, these records, in addition to serving as an essential component for patient care, can be utilized for forensic and legal purposes [14]. Therefore, the exposure of undergraduate students to forensic odontology underscores the professional responsibility for precise dental documentation. It also emphasizes the pivotal role of accurate dental records in the identification of individuals, as well as in cases involving abuse, violence, or trauma, including instances of child abuse [8,15]. Formal teaching in forensic odontology has existed for over 100 years and constitutes an integral part of undergraduate dental training [8,16]. However, research conducted among dental students indicates insufficient knowledge and a lack of practical experience [17]. Delivering comprehensive undergraduate training will empower future dentists to actively contribute to the safeguarding of vulnerable individuals in cases of abuse and the identification of victims in mass disasters [18].

The Republic of Cyprus, a member country of the European Union, lacked a tertiary dental institution, and no educational programs in the field of forensic odontology were offered for local dentists. Consequently, forensic odontology was primarily conducted by coroners, with occasional assistance sought from other countries, predominantly Greece. In 2017, European University Cyprus (EUC) admitted its inaugural cohort into the newly established School of Medicine’s Department of Dentistry, which later, in 2022, became the independent School of Dentistry. Currently, this is the sole accredited dental institution in Cyprus. The Bachelor of Surgery (BDS) program includes an elective course “Legal and Forensic Dentistry” in its 4th year curriculum.

The aim of the present study was to investigate knowledge, attitudes, and practice relevant to forensic odontology among undergraduate dental students and faculty members of the School of Dentistry in European University Cyprus. The null hypotheses is that there are no significant variations in the levels of knowledge, attitudes, and practices related to forensic odontology among undergraduate dental students and faculty members at the School of Dentistry, European University Cyprus. This study was approved by the Institutional Committee on Bioethics and Ethics of the European University Cyprus.

## 2. Materials and Methods

The data presented here is derived from a cross-sectional survey conducted among 382 undergraduate dental students and 71 full-time and part-time faculty members, all of whom were enrolled students or teaching staff at the EUC School of Dentistry at the time of the survey.

After obtaining consent to participate, two distinct questionnaires, in English, were administered via e-mail—one for students and one for faculty. Each questionnaire comprised two parts. The Faculty Questionnaire consisted of Part I, consisting of 5 demographic questions, and Part II, consisting of 15 study questions related to knowledge, attitudes and practices in forensic dentistry. On the other hand, the Student Questionnaire included Part I, consisting of 3 demographic questions, and Part II, consisting of 15 study questions. Notably, the only difference in the study questions between the student and faculty questionnaire was in question 11; faculty members were asked whether they maintain dental records in their clinics, while students were asked if they believed meticulous dental record-keeping is a significant component of quality patient care. All questions were multiple choice, except for question 15, which was open/essay-type. In this question, participants were invited to provide suggestions to increase awareness of forensic odontology/dentistry among dental students, dentists, and other health professionals.

The questionnaires utilized in this study were adapted from Abdul et al. [17] and Jayakumar et al. [11] to ensure that the selected questions are relevant and applicable, capturing insights specific to the context of the EUC School of Dentistry. The rationale behind the selection of these specific questionnaires lies in their comprehensiveness, effectively encompassing the key aspects of forensic odontology, and their previous application involving dental students. To tailor the questionnaires to our study’s specific requirements, we extended the scope to include faculty members, necessitating modifications to certain questions, such as those related to dental records.

The survey, hosted on Google Forms and compliant with GDPR, could be completed in approximately 5 min, with the survey link accessible throughout November 2022.

Survey data were summarized through calculating percentages for all variables. Kendall’s *tau* test used to measure the ordinal association between two measured quantities was utilized to assess the correlation of faculty responses with biological sex, age group, employment type, number of courses taught and the highest educational qualification. For student responses, the correlation was analyzed with biological sex, age group and year of study. The association of faculty and student responses with the aforementioned factors were tested using the *χ*^2^ test to examine whether the two categorical variables are independent in influencing the values. Additionally, for statistically significant associations, Cramer’s *V* was calculated to measure the strength of association. Significance levels (*p*-values) were predetermined at *α* = 0.05 (*p* ≤ 0.05) and were estimated using the Monte-Carlo simulation method [19]. All analyses were conducted using SPSS v.26 (IBM Corp, Armonk, New York 10504-1722, USA).

## 3. Results

The survey was completed by 47 faculty members (66.2% response rate) and 304 students (80% response rate). The demographic characteristics of the respondents are presented in Table 1. While faculty answers were not in general correlated with demographic characteristics, some student responses were significantly correlated with biological sex, age and year of study, reflecting differing associations across the student body.

The answers of faculty members and students to the survey questions are presented in Table 2.

Many respondents indicated the utility of forensic odontology in criminal and deceased individual identification. However, a notable proportion of students (35.2%) were unaware that forensic odontology constitutes a branch of dentistry. Additionally, a small percentage of faculty members (6.4%) and students (13.2%) lacked awareness that teeth can serve as a source of DNA.

While a significant majority of faculty (85.1%) and students (81.6%) acknowledged that dental age can be determined through eruption patterns and calcification, a considerable number included biochemical and/or histological methods in their responses. The importance of dental records in determining the age and biological sex of a deceased person in mass disasters was emphasized by most faculty members (78.7%) and students (78%).

Many faculty members (61.7%) and students (61.5%) did not correctly identify the study of lip prints in forensic odontology as cheiloscopy. However, a majority (63.5% of students and 85.1% of faculty) were aware of the significance of bite mark patterns.

Interestingly, a substantial portion of faculty (74.5%) and students (86.5%) had no knowledge about forensic odontology. Despite this, only 66% of faculty members expressed willingness to participate in workshops and seminars on the subject.

Nearly 79% of the faculty maintained records in their clinics, while 78% of students believed that dental record-keeping is a significant aspect of quality patient care. A significant percentage of faculty members (95.7%) asserted that child abuse cases can be identified through physical injuries, scars, clothing, and behavioral changes, with a majority of students (84.5%) sharing this perspective.

Regarding actions in child abuse cases, 80.9% of the faculty favored reporting to the police, while 19.1% preferred reporting to the parents. Students’ responses mirrored this pattern. Furthermore, a notable proportion of faculty (63.8%) and students (56.9%) were aware that a dentist can testify as an expert witness in a court of law with forensic dental evidence.

The correlations of faculty and student answers to the second part of the survey with the demographic characteristics are presented in Table 3 and Table 4.

Positive, weak, and statistically significant relationships were identified between faculty members’ educational backgrounds and their responses to the questions “Can teeth serve as a source of DNA?” and “Do you maintain dental records in your clinic?” as detailed in Table 3.

In the case of students, positive, weak, and statistically significant correlations were observed between their biological sex and responses to questions such as “Is forensic dentistry useful in identifying criminals and the deceased?” and “Are you interested in participating in workshops and seminars in forensic odontology?” Additionally, a negative, weak, and statistically significant relationship was noted between biological sex and the awareness of forensic odontology as a branch in Dentistry. Furthermore, students’ age groups exhibited negative, weak, and statistically significant relationships with responses to questions like “Do you know about forensic odontology as a branch in dentistry?” and “Can teeth serve as a source of DNA?”. Similarly, students’ year of study showed negative, weak, and statistically significant relationships with answers to questions like “Do you know about forensic odontology as a branch in dentistry?” and “Is forensic dentistry useful in identifying criminals and the deceased?” (Table 4).

Table 5 presents the association of faculty members’ responses with biological sex, age group, employment type, number of courses taught, and educational background (highest qualification). However, according to the results of the χ^2^ test, no statistically significant associations were observed.

The association of student answers with biological sex, age group and year of study is presented in Table 6.

The χ^2^ test results revealed an association between biological sex and responses to the questions “Is forensic dentistry useful in identifying criminals and the deceased?” (*p* = 0.033; Cramer’s V 0.101) and “Are you interested in participating in workshops and seminars in forensic odontology?” (*p* < 0.001; Cramer’s V 0.224). In both instances, statistically and significantly more positive answers were noted from females (Table 7).

Furthermore, a statistically significant association was noted between students’ age groups and responses to the question “Do you know about forensic odontology as a branch in dentistry?” (*p* = 0.008; Cramer’s V 0.213). The majority of students aged between 22 and 28 years old were knowledgeable about forensic odontology as a branch in dentistry, contrasting with younger students aged 18 to 21 and those older than 28 years old (Table 8).

Lastly, statistically significant associations were identified between students’ year of study and responses to the questions “Do you know about forensic odontology as a branch in dentistry?” (*p* < 0.001; Cramer’s V0.298), “What is the study of lip prints in forensic dentistry called?” (*p* = 0.012; Cramer’s V 0.176), and “Are you interested in participating in workshops and seminars in forensic odontology?” (*p* < 0.001; Cramer’s V 0.283). With an increase in the year of study, more students demonstrated awareness of forensic odontology as a branch in dentistry (Table 9).

As per the findings of the χ^2^ test (Table 10), a majority of students in the first, third, and fourth years of study lacked knowledge about the term for lip prints in forensic dentistry. Conversely, most students in the second and fifth years of study were aware that it is called cheiloscopy.

Several students in the first and fourth years of study expressed interest in participating in workshops and seminars in forensic odontology. Conversely, nearly all students in the second, third, and fifth years demonstrated an interest in participating in such workshops and seminars (Table 11).

## 4. Discussion

Each year, various natural disasters, accidents, and malicious acts result in numerous deaths, leaving behind unidentified victims [20]. The oral health profession plays a crucial role in supporting forensic investigations for victim identification. Forensic odontology, a specialized branch dedicated to this purpose, is invaluable in human identification processes, primarily due to the distinct nature of oral hard tissue anatomical features [1,21]. For oral health professionals to contribute effectively, awareness of this dentistry branch is essential within the dental community.

In our study within Cyprus, we assessed awareness, knowledge, attitudes, and practices related to forensic odontology among faculty and undergraduate students at the School of Dentistry, European University Cyprus. Established and accredited in the European Union in 2017, this institution represents the sole academic body in the Republic of Cyprus offering a Bachelor of Dental Surgery program, including an elective “Legal and Forensic Dentistry” course in the fourth academic year.

A significant portion of our respondents, with 87% of faculty and 65% of students, demonstrated awareness of forensic odontology as a dental specialization. This high level of awareness suggests a positive trend, indicating that the dental community in Cyprus recognizes the importance of forensic odontology in contributing to human identification processes. Our findings are consistent with studies conducted in Saudi Arabia, which reported awareness levels ranging from 62.5% to 78.4% [6,22]. However, our study surpasses the awareness levels observed among Indian dentists [23]. This variation in awareness levels across different regions may be influenced by cultural factors, educational emphasis, and the prevalence of forensic odontology within the respective healthcare systems. Furthermore, the awareness levels among students in our study closely align with results from analogous studies [17,23,24].

Eighty-five percent of our student respondents recognized teeth as a DNA source, surpassing results from Abdul et al., which ranged from 40% to 75% across different educational levels [17]. This heightened awareness among students may be attributed to the specific curriculum, highlighting the success of the educational initiatives in conveying crucial aspects of forensic odontology.

Faculty awareness in our study stood at 94%, mirroring Sahni et al., where 95% of 200 dental faculty members acknowledged this fact [8]. The high level of faculty awareness is encouraging, as it indicates that educators, who play a pivotal role in shaping future dental professionals, are well-informed about the role of teeth as a DNA source. Furthermore, our study revealed a positive, weak, and statistically significant correlation between faculty’s academic qualifications and their awareness levels, also found in the question regarding maintaining dental records in their private clinic. This correlation suggests that higher academic qualifications may contribute to a more nuanced understanding of forensic odontology, emphasizing the importance of continuous education for dental professionals. A majority of our faculty (97.9%) and students (88.8%) agreed on the utility of forensic odontology in identifying both criminals and deceased individuals. These statistics are comparable to a study in Saudi Arabia, reporting 95% awareness among students [17].

Regarding awareness of methods for dental age identification in children and adults, our results (15% for students and 13% for faculty who did not know how to identify/estimate dental age) appeared modest compared to the 25% awareness recorded by Abdul et al. [17]. This discrepancy may indicate a need for additional educational emphasis on age identification methods in the course syllabus. While DNA profiling, fingerprints, anthropometric data, and dental records remain predominant methods for human identification, supplementary techniques such as cheiloscopy, palatoscopy, and other odontometric measurements yield reliable outcomes when systematically employed. Regarding cheiloscopy, which studies the unique patterns of “lip prints”, i.e., the elevations and depressions of the external surface of the lips, our results (38.3% for faculty and 38.5% for students) align closely with others’ findings among dental professionals and dental students [17]. Notably, 51% of faculty answered “I do not know how the study of lip prints is called”, whereas this percentage was 42.8% for students. This discrepancy in awareness levels indicates a potential area for improvement in educating faculty members about specific forensic odontology techniques.

We observed varying levels of awareness regarding the significance of bite mark patterns in teeth with of 85.1% among faculty and 63.5% among students. Studies from India and Pakistan reported ignorance levels of 32% and 48%, respectively [23,25], contrasting our findings. Conversely, a study in Saudi Arabia highlighted recognition levels in forensic odontology importance, with 87.5% among postgraduate students, 50% among graduate students, and 27.3% among undergraduates [17].

Awareness sources varied between students and faculty, with students relying on the internet and faculty on books and lectures. Both groups self-assessed their knowledge as limited, a sentiment echoed in other studies [17,26]. This self-assessment indicates a recognition of the complexity of forensic odontology and a desire for further education. The inclination to attend forensic odontology workshops and seminars was evident, with 66% of faculty expressing interest (lower than in other studies) and 85.2% of students expressing interest (similar to other studies) [8,12,17,25,26].

Patient records, essential for quality care and legal prerequisites, also serve as valuable tools in forensic odontology [27]. Our findings indicate that a significant 80% of faculty maintain these records, consistent with the 90% reported by Savić Pavičin et al. in Croatia [15]. In another study conducted by Preethi et al., it was found that only 12% maintained a complete dental record, 21% did not maintain any record, and 93% of the dentists did not preserve a record for more than 7 years [12]. Astekar et al. reported that only 38% of dentists retain records, whereas 62% did not maintain any record [28]. In another study, Waleed et al. observed that accurate maintenance of dental records is more prominent among dental students compared to private practitioners [29]. In a recent study in India among postgraduate students and practicing clinicians, a very high percentage, 97%, of the participants maintained dental records [30]. In our study, students expressed their belief that meticulous dental record keeping is a significant component of quality patient care, with 78% indicating this perspective. This may reflect the strong encouragement by the School for proper record keeping.

Addressing the grave issue of child abuse, a serious social problem increasing at an alarming rate globally, early identification is of paramount importance [8]. In our study, high awareness levels were observed, with nearly 96% of faculty and 85% of students agreeing that child abuse cases can be identified by physical injuries, scars, clothing, and behavioral changes. These figures contrast significantly with Abdul’s findings, where only 25% of respondents recognized these indicators and 12.5% did not know how to identify child abuse [17], when in our study the same measure was 5.9%.

In addressing the issue of child abuse reporting, our findings offer some optimism. Among our participants, 81% of faculty and 85% of students expressed willingness to report suspected child abuse to the police. In comparison, a distinct study reported a considerably lower percentage of 25% taking similar action. Disturbingly, 3% of our student participants indicated they would abstain from any action, though this represents an improvement from the 12.5% reported in Abdul’s Riyadh-based study [17]. A survey from India indicated that 41% of dental teaching staff would prefer to report suspicions directly to the child’s parents [8]. In contrast, our data show lower figures, with only 20% of faculty and 12% of students choosing this course of action. It is important to highlight the absence of a dedicated on-site social worker at our institution to promptly address child abuse suspicions, which would arguably be a preferred initial step before police or family involvement. The intricate nature of domestic violence, often occurring within family confines, warrants careful handling. Directly notifying parents without comprehensive case assessment might exacerbate the situation rather than alleviate it.

On another front, our study revealed that 64% of faculty and 57% of students were aware of the role dentists play in the legal system, specifically in providing expert testimonies and presenting forensic dental evidence in court. In contrast, Abdul et al.’s study exhibited a higher awareness level at 85% [17].

Variations in the awareness levels observed in our study compared to others may stem from several factors: Different regions and cultures may prioritize or emphasize certain aspects of forensic odontology differently. Variances in dental education systems and curricula can influence the exposure and understanding of forensic odontology. Our study focused on a specific dental school in Cyprus, and differences may arise due to the unique educational context of that institution. The timing of the study may also matter, as changes in awareness can occur due to evolving educational programs, advancements in technology, or increased media coverage. Differences in the demographic composition of study participants, such as age, academic background, or clinical experience, can contribute to variations in awareness. Our study focused on a specific group within one dental school, and this group’s characteristics may differ from those in other studies. Regional variations in legal frameworks and professional guidelines regarding forensic odontology may also influence awareness levels. Finally, sensitivity towards issues such as child abuse may vary across cultures. This can influence both the awareness of indicators and the willingness to report suspicions.

Forensic odontology, while well-explored globally, remains under-researched in Cyprus. To date, its application in Cyprus has primarily been via coroners, with government dentists consulted on a need basis. A poignant example that underscores its importance is the Helios Airways Flight 522 tragedy in 2005, where 121 passengers and crew were killed and burnt. Dental professionals played an instrumental role in victim identification. In that case, dental records were requested form the victims’ dentists in Cyprus and Greece. Forensic odontology specialists from the Department of Dentistry of the School of Health Sciences of the National and Kapodistrian University of Athens, worked on identification.

This study addresses a gap in forensic odontology research within Cyprus, where formal education and training in this field have been limited. By evaluating awareness, knowledge, attitudes, and practices at the European University Cyprus School of Dentistry, the study provides insights into the current state of forensic odontology among faculty and undergraduate dental students. The establishment of the School of Dentistry and the introduction of the “Legal and Forensic Dentistry” elective course represent significant strides in addressing the educational void in Cyprus. An argument could be made for rendering “Legal and Forensic Dentistry” to a mandatory course, ensuring universal student exposure.

The incorporation of forensic odontology into the curriculum of dental schools is particularly vital in today’s context and it provides substantial knowledge in disaster victim identification addressing cases of missing persons, unidentified individuals, child abuse, and age estimation [31,32]. However, it is noteworthy that the subject of forensic odontology is not currently included in the basic dental training outlined in Directive 2005/36/EC of the European Parliament and of the Council of 7 September 2005 on the recognition of professional qualifications—Section 4 [33]. Consideration should be given to incorporating forensic odontology into the basic dental training curriculum to align with the evolving demands of the field and enhance the professional qualifications of dental practitioners.

The findings not only provide a baseline understanding, but also propose targeted interventions to enhance education, including seminars and workshops for faculty. Additionally, the study highlights the role dentists can play in legal contexts, particularly in cases of child abuse, offering valuable insights for further professional development and curriculum refinement in Cyprus. Policymakers could consider incorporating forensic odontology modules or courses through the Cyprus Dental Council and local Dental Associations to ensure comprehensive training for dentists in the community. Awareness campaigns could inform local dentists about the importance of forensic odontology. The study also underscores the importance of meticulous dental record-keeping, prompting policymakers to consider developing standardized guidelines for dental record-keeping practices, ensuring uniformity and accuracy in documentation, both for patient care and forensic purposes.

Limitations of this study include focus solely on a single dental school in Cyprus, which may limit the generalization of findings to the broader population of dental professionals in the country. Additionally, the sample size, while reasonable, may not fully represent the diversity of perspectives within the dental community. The reliance on self-reported data introduces the potential for recall bias and social desirability bias, impacting the accuracy of reported awareness and practices. The cross-sectional design provides a snapshot of awareness at a specific point in time but does not capture changes or trends over time. Response bias may be present, as individuals who chose to participate may differ in their awareness levels from those who chose not to participate. The study does not extensively explore the cultural context and its potential impact on awareness and attitudes toward forensic odontology, leaving room for further investigation. Addressing these limitations in future research would enhance the robustness and applicability of studies in forensic odontology within the Cypriot context.

## 5. Conclusions

The survey highlighted a robust awareness of forensic odontology among respondents. While the faculty demonstrates comprehensive understanding, there is a pronounced need to enhance their inclination towards attending relevant seminars. It is paramount to emphasize improved record-keeping practices for potential forensic applications.

## Figures and Tables

**Table 1:** Faculty members (n = 47) and students (n = 304) demographic characteristics.

Faculty [n (%)]	Students [n (%)]
**Biological sex** 25 (53.2%) Male 22 (46.8%) Female	**Biological sex** 129 (42.4%) Male 172 (56.6%) Female 3 (1.0%) Prefer not to say
**Age group** 10 (21.3%) 26–35 years-old 15 (31.9%)36–45 years-old 12 (25.5%) 46–55 years-old 10 (21.3%) >55 years-old	**Age group** 4 (1.3%) <18 163 (53.6%) 18–21 years-old 100 (32.9%) 22–25 years-old 24 (7.9%) 26–28 years-old 13 (4.3%) >28 years-old
**Employment type** 12 full-time (25.5%) 35 part-time (74.5%)	**Year of study** 59 (19.4%) year 1 students 61 (20.1%) year 2 students 86 (28.3%) year 3 students 51 (16.8%) year 4 students 45 (14.8%) year 5 students 2 (0.7%) already hold a Dental Degree from another University and study in year 3,4,5
**Number of courses taught** 19 (40.4%) one course taught 17 (36.2%) two courses taught 5 (10.6%) three courses taught 6 (12.8%) four courses taught	
**Educational background (highest qualification)** 19 (40.4%) possess a PhD 22 (46.8%) possess a Master 6 (12.8%) possess a Bachelor Degree	

**Table 2:** Answers of faculty members (n = 47) and students (n = 304) to the survey questions.

Survey Questions	Answers	Faculty [n (%)]	Students [n (%)]
**Are you aware that the forensic odontology is a branch of dentistry?**	Yes	41(87.2%)	197 (64.8%)
	No	6 (12.8%)	107 (35.2%)
**Can teeth serve as source of DNA?**	Yes	44 (93.6%)	257 (84.5%)
	No	0 (0%)	7 (2.3%)
	I do not know	3 (6.4%)	40 (13.2%)
How do you identify the dental age in children and adults? *	Eruption patterns and calcification	40 (85.1%)	248 (81.6%)
	Histological methods	24 (51.1%)	148 (48.7%)
	Biochemical methods	15 (31.9%)	94 (30.9%)
	I do not know	6 (12.8%)	45 (14.8%)
How will you identify a deceased person’s age and gender in mass disasters? *	Reconstruct the fragmented deceased body	18 (38.3%)	111 (36.5%)
	Dental records	42 (89.4%)	221 (72.7%)
	Fingerprints	17 (36.2%)	98 (32.2%)
	I do not know	5 (10.6%)	56 (18.4%)
**Is forensic dentistry useful in identifying criminals and dead people?**	Yes	46 (97.9%)	270 (88.8%)
	No	0 (0%)	2 (0.7%)
	I do not know	1 (2.1%)	32(10.5%)
** What is the study of lip prints in forensic dentistry called? **	Lipology	1 (2.1%)	39 (12.8%)
	Cheiloscopy	18 (38.3%)	117 (38.5%)
	Dermatoglyphics	4 (8.5%)	18 (5.9%)
	I do not know	24 (51.1%)	130 (42.8%)
**Are you aware of the significance of bite mark pattern of teeth?**	Yes No	40 (85.1%) 7 (14.9%)	193 (63.5%) 111 (36.5%)
What is the source of your knowledge about forensic dentistry? *	Books	19 (40.4%)	59 (19.4%)
	Internet	18 (38.3%)	191 (62.8%)
	Workshops, seminars, lectures	29 (61.7%)	101 (33.2%)
	I do not have knowledge	7 (14.9%)	97(31.9%)
**Do you think your knowledge and awareness about forensic odontology is enough?**	Yes	4 (8.5%)	13 (4.3%)
	No	35 (74.5%)	263 (86.5%)
	I do not know	8 (17%)	28 (9.2%)
** Are you interested to participate in workshops and seminars in forensic odontology? **	Yes	31 (66%)	259 (85.2%)
	No	16 (34%)	45 (14.8%)
**Do you maintain dental records in your clinic? ^1^**	Yes	37 (78.7%)	
	No	4 (8.5%)	
	N/A	6 (12.8%)	
**Do you think meticulous dental record keeping is a significant component of quality patient care? ^2^**	Yes		237 (78%)
	No		9 (3%)
	Maybe		58 (19.1)
**How will you identify physical/neglected/sexual/psychological abuse of a child?**	Physical injuries	0 (0%)	15 (4.9%)
	Behavioral changes	1 (2.1%)	10 (3.3%)
	Clothing	0 (0%)	3 (1%)
	Any scars	1 (2.1%)	1 (0.3%)
	All the above	45 (95.7%)	257 (84.5%)
	I do not know	0 (0%)	18 (5.9%)
** What action would you take, if you identify child abuse? **	Inform police	38 (80.9%)	258 (84.9%)
	Inform parents	9 (19.1%)	36 (11.8%)
	Take no action	0 (0%)	10 (3.3%)
**Are you aware that you can testify as an expert witness in the court to present forensic dental evidence?**	Yes	30 (63.8%)	173 (56.9%)
	No	10 (21.3%)	85 (28%)
	N/A	7 (14.9%)	46 (15.1%)

* Multiple answers could be selected; ^1^ This question was addressed only to faculty; ^2^ This question was addressed only to students.

**Table 3:** Correlations of faculty answers to survey questions with demographic characteristics *.

	Biological Sex	Age Group	Employment Type	Number of Courses Taught	Educational Background
**Are you aware that the forensic odontology is a branch of dentistry?**	tau = 0.103 *p* = 0.484	tau = 0.043 *p* = 0.770	tau = 0.078 *p* = 0.598	tau = 0.021 *p* = 0.879	tau = −0.111 *p* = 0.431
**Can teeth serve as source of DNA?**	tau = 0.071 *p* = 0.632	tau = 0.136 *p* = 0.357	tau = −0.153 *p* = 0.300	tau = 0.181 *p* = 0.187	**tau = 0.284** ***p* = 0.045**
**Is forensic dentistry useful in identifying criminals and dead people?**	tau = 0.138 *p* = 0.348	tau = 0.077 *p* = 0.603	tau = −0.086 *p* = 0.558	tau = −0.151 *p* = 0.272	tau = 0.074 *p* = 0.599
** What is the study of lip prints in forensic dentistry called? **	tau = 0.032 *p* = 0.822	tau = −0.020 *p* = 0.885	tau = 0.040 *p* = 0.776	tau = 0.135 *p* = 0.306	*tau* = −0.075, *p* = 0.582
**Are you aware of the significance of bite mark pattern of teeth?**	tau = −0.087 *p* = 0.557	tau = −0.211 *p* = 0.135	tau = 0.029 *p* = 0.843	tau = 0.164 *p* = 0.233	*tau* = −0.009, *p* = 0.948
** Do you think your knowledge and awareness about forensic odontology is enough? **	tau = 0.239 *p* = 0.096	tau = −0.007 *p* = 0.959	tau = −0.101 *p* = 0.482	tau = 0.203 *p* = 0.129	*tau* = −0.190, *p* = 0.168
** Are you interested to participate in workshops and seminars in forensic odontology? **	tau = 0.134 *p* = 0.363	tau = 0.264 *p* = 0.074	tau = 0.197 *p* = 0.181	tau = 0.113 *p* = 0.410	*ta*u = 0.031 *p* = 0.824
** Do you maintain dental records in your clinic? **	tau = 0.187 *p* = 0.194	tau = −0.113 *p* = 0.434	tau = 0.032 *p* = 0.824	tau = −0.083 *p* = 0.537	**tau = 0.317** ***p* = 0.022**
** How will you identify physical/neglected/sexual/psychological abuse of a child? **	tau = 0.197 *p* = 0.180	tau = 0.109 *p* = 0.457	tau = −0.123 *p* = 0.403	tau = 0.054 *p* = 0.695	tau = −0.065 *p* = 0.644
** What action would you take, if you identify child abuse? **	tau = 0.131 *p* = 0.373	tau = −0.011 *p* = 0.939	tau = 0.087 *p* = 0.555	tau = 0.075 *p* = 0.585	tau = 0.019 *p* = 0.894
** Are you aware that you can testify as an expert witness in the court to present forensic dental evidence? **	tau = 0.005 *p* = 0.970	tau = −0.052 *p* = 0.715	tau = −0.251 *p* = 0.076	tau = −0.024 *p* = 0.854	tau = 0.061 *p* = 0.652

* Questions allowing single answers only. Statistically significant *p*-values in bold.

**Table 4:** Correlations of student answers to survey questions with demographic characteristics *.

	Biological Sex	Age Group	Year of Study
**Are you aware that the forensic odontology is a branch of dentistry?**	tau = −0.020 *p* = 0.723	**tau = −0.144** ***p* = 0.009**	**tau = −0.264** ***p* < 0.001**
**Can teeth serve as source of DNA?**	tau = 0.013 *p* = 0.821	**tau = −0.135** ***p* = 0.045**	tau = −0.083 *p* = 0.104
**Is forensic dentistry useful in identifying criminals and dead people?**	**tau = 0.126** ***p* = 0.027**	**tau = −0.135** ***p* = 0.013**	**tau = −0.102** ***p* = 0.048**
** What is the study of lip prints in forensic dentistry called? **	tau = 0.039 *p* = 0.467	tau = −0.030 *p* = 0.555	**tau = −0.158** ***p* = 0.001**
**Are you aware of the significance of bite mark pattern of teeth?**	tau = −0.030 *p* = 0.596	tau = −0.009 *p* = 0.870	tau = −0.047 *p* = 0.366
** Do you think your knowledge and awareness about forensic odontology is enough? **	tau = 0.105 *p* = 0.061	tau = −0.064 *p* = 0.238	tau = −0.041 *p* = 0.414
** Are you interested to participate in workshops and seminars in forensic odontology? **	**tau = 0.207** ***p* < 0.001**	tau = −0.016 *p* = 0.765	tau = −0.058 *p* = 0.257
**Do you think meticulous dental record keeping is a significant component of quality patient care?**	tau = 0.077 *p* = 0.173	**tau = −0.129** ***p* = 0.017**	tau = −0.076 *p* = 0.136
** How will you identify physical/neglected/sexual/psychological abuse of a child? **	**tau = 0.123** ***p* = 0.028**	tau = −0.099 *p* = 0.062	tau = −0.071 *p* = 0.155
** What action would you take, if you identify child abuse? **	tau = 0.077 *p* = 0.173	tau = 0.065 *p* = 0.230	tau = −0.028 *p* = 0.576
** Are you aware that you can testify as an expert witness in the court to present forensic dental evidence? **	tau = 0.024 *p* = 0.644	**tau = −0.128** ***p* = 0.014**	tau = −0.038 *p* = 0.446

* Questions allowing single answers only. Statistically significant *p*-values in bold.

**Table 5:** Statistical significance difference levels of the χ^2^ test associations of faculty answers to survey questions with demographic characteristics *.

	Biological Sex	Age Group	Employment Type	Number of Courses Taught	Educational Background
**Are you aware that the forensic odontology is a branch of dentistry?**	0.670	1.000	0.678	0.812	0.717
**Can teeth serve as source of DNA?**	1.000	0.589	0.560	0.382	0.095
**Is forensic dentistry useful in identifying criminals and dead people?**	1.000	1.000	1.000	1.000	1.000
** What is the study of lip prints in forensic dentistry called? **	0.855	0.731	0.206	0.248	0.171
**Are you aware of the significance of bite mark pattern of teeth?**	0.690	0.155	0.842	0.539	1.000
** Do you think your knowledge and awareness about forensic odontology is enough? **	0.273	0.504	0.739	0.285	0.143
** Are you interested to participate in workshops and seminars in forensic odontology? **	0.538	0.131	0.289	0.583	1.000
** Do you maintain dental records in your clinic? **	1.000	1.000	0.559	0.055	0.060
** How will you identify physical/neglected/sexual/psychological abuse of a child? **	1.000	1.000	1.000	0.418	0.788
** What action would you take, if you identify child abuse? **	0.470	1.000	0.674	0.227	0.592
** Are you aware that you can testify as an expert witness in the court to present forensic dental evidence? **	1.000	1.000	0.693	1.000	0.391

* Questions allowing single answers only.

**Table 6:** Statistical significance difference levels of the χ^2^ test associations of student answers to survey questions with demographic characteristics *.

	Biological Sex	Age Group	Year of Study
**Are you aware that the forensic odontology is a branch of dentistry?**	0.807	**0.008**	**<0.001**
**Can teeth serve as source of DNA?**	0.198	0.435	0.933
**Is forensic dentistry useful in identifying criminals and dead people?**	**0.033**	0.163	0.103
** What is the study of lip prints in forensic dentistry called? **	0.441	0.857	**0.012**
**Are you aware of the significance of bite mark pattern of teeth?**	0.630	0.902	0.117
** Do you think your knowledge and awareness about forensic odontology is enough? **	0.080	0.606	0.756
** Are you interested to participate in workshops and seminars in forensic odontology? **	**<0.001**	0.122	**<0.001**
**Do you think meticulous dental record keeping is a significant component of quality patient care?**	0.431	0.118	0.212
** How will you identify physical/neglected/sexual/psychological abuse of a child? **	0.067	0.240	0.065
** What action would you take, if you identify child abuse? **	0.453	0.526	0.148
** Are you aware that you can testify as an expert witness in the court to present forensic dental evidence? **	0.706	0.506	0.981

* Questions allowing single answers only. Statistically significant *p*-values in bold.

**Table 7:** Distribution of answers to the questions “Is forensic Dentistry useful in identifying criminals and the dead people?” and “Are you interested to participate in workshops and seminars?” according to the students’ biological sex.

	Is Forensic Dentistry Useful in Identifying Criminals and the Dead People?				Are You Interested to Participate in Workshops and Seminars?		
Biological Sex	Yes	No	I Do not Know	Total	Yes	No	Total
**Female**	159 (92.4%)	1 (0.6%)	12 (7.0%)	172 (100%)	158 (91.9%)	14 (8.1%)	172 (100%)
**Male**	108 (83.7%)	1 (0.8%)	20 (15.5%)	129 (100%)	98 (76.0%)	31 (24.0%)	129 (100%)
**Total**	267 (88.7%)	2 (0.7%)	32 (10.6%)	301 (100%)	256 (85.0%)	45 (15.0%)	301 (100%)

**Table 8:** Distribution of the answers for the question “Do you know about forensic odontology as a branch in dentistry?” according to the students’ age group.

Age Group	Yes	No	Total
18–21	92 (56.3%)	71 (43.6%)	163 (100%)
22–25	73 (73.0%)	27 (27.0%)	100 (100%)
26–28	20 (83.3%)	4 (16.7%)	24 (100%)
>28	8 (61.5%)	5 (38.5%)	13 (100%)
Total	193 (64.3%)	107 (35.7%)	300 (100%)

**Table 9:** Distribution of the answers for the following question “Do you know about forensic odontology as a branch in dentistry?” according to the students’ year of study.

Year of Study	Yes	No	Total
1st	25 (42.4%)	34 (57.6%)	59 (100%)
2nd	34 (55.7%)	27 (44.3%)	61 (100%)
3rd	59 (68.6%)	27 (31.4%)	86 (100%)
4th	40 (78.4%)	11 (21.6%)	51 (100%)
5th	37 (82.2%)	8 (17.8%)	45 (100%)
Total	195 (64.6%)	107 (35.4%)	304 (100%)

**Table 10:** Distribution of the answers for the following question “What is the study of lip prints in forensic Dentistry called?” according to the students’ year of study.

Year of Study	Dermatoglyphics	Cheiloscopy	Lipology	I Do Not Know	Total
1st	2 (3.4%)	14 (23.7%)	13 (22.0%)	30 (50.8%)	59 (100%)
2nd	4 (6.6%)	27 (44.3%)	9 (14.8%)	21 (34.4%)	61 (100%)
3rd	4 (4.7%)	32 (37.2%)	8 (9.3%)	42 (48.8%)	86 (100%)
4th	3 (5.9%)	17 (33.3%)	4 (7.8%)	27 (52.9%)	51 (100%)
5th	5 (11.1%)	25 (55.6%)	5 (11.1%)	10 (22.2%)	45 (100%)
Total	18 (6.0%)	115 (38.1%)	130 (43.0%)	39 (12.9%)	304 (100%)

**Table 11:** Distribution of the answers for the following question “Are you interested to participate in workshops and seminars in forensic odontology?” according to the students’ year of study.

Year of Study	Yes	No	Total
1st	44 (74.6%)	15 (25.4%)	59 (100%)
2nd	53 (86.9%)	8 (13.1%)	61 (100%)
3rd	83 (96.5%)	3 (3.5%)	86 (100%)
4th	36 (70.6%)	15 (29.4%)	51 (100%)
5th	41 (91.1%)	4 (8.9%)	45 (100%)
Total	257 (85.1%)	45 (14.9%)	304 (100%)

## Data Availability

Data is contained within the article.
